# A Descriptive Study on Clinical Pulmonary Infection Score (CPIS) in Early Identification of Hospital-Acquired Pneumonia (HAP) Among Critically Ill Patients in a Tertiary Care Teaching Hospital

**DOI:** 10.7759/cureus.88841

**Published:** 2025-07-27

**Authors:** Shrabani Mondal, Ferganzia Jubilson, Sagar Sinha

**Affiliations:** 1 Nursing Practice: Critical Care, MGM New Bombay College of Nursing, Navi Mumbai, IND; 2 Nursing, MGM New Bombay College of Nursing, Navi Mumbai, IND; 3 Emergency Medicine, MGM Medical College and Hospital, MGM Institute of Health Sciences, Navi Mumbai, IND

**Keywords:** clinical pulmonary infection score, critically ill, hospital acquired pneumonia, intensive care unit, tertiary care teaching hospital

## Abstract

Introduction: Hospital-acquired pneumonia (HAP), including ventilator-associated pneumonia (VAP) and healthcare-associated pneumonia (HCAP), occurs more than 48 hours after hospital admission and is often associated with multidrug-resistant (MDR) pathogens. Prompt and accurate diagnosis is critical, relying on clinical criteria such as fever, leukocytosis, purulent sputum, and new infiltrates on imaging, along with respiratory cultures to identify causative organisms. Empirical antibiotic therapy should be initiated early, targeting likely pathogens, but must be refined through de-escalation once culture results are available. This approach helps reduce the risk of resistance, minimizes unnecessary antibiotic use, and improves patient outcomes in healthcare settings.

Materials and methods: The research was conducted within a tertiary intensive care unit (ICU), focusing on a cohort of 225 adult patients who were hospitalized for a duration exceeding 48 hours. Participants were selected using a purposive sampling method. Data collection involved observational techniques, utilizing validated assessment instruments that exhibited a strong internal consistency, evidenced by a Cronbach’s alpha of 0.829. The data analysis employed Chi-square statistics to investigate associations among the variables studied. This methodological framework ensured robust findings and yielded valuable insights into clinical patterns and outcomes pertinent to patients in critical care environments.

Results: A study conducted on ICU patients revealed that a substantial 85.3% exhibited a low Clinical Pulmonary Infection Score (CPIS), characterized by moderate pyrexia and minimal respiratory secretions. In contrast, 14.7% of patients were classified as high-risk, demonstrating pronounced inflammatory responses and specific radiographic abnormalities. Analysis using Kendall’s tau_b established a positive correlation between CPIS scores and the incidence of HAP. Among the cohort of 225 critically ill patients, those identified as high CPIS risk had a significantly elevated occurrence of HAP (p=0.001), underscoring the utility of CPIS as a robust predictive indicator for pneumonia risk in this population.

Conclusion: The CPIS serves as an effective prognostic tool for the early identification of HAP in critically ill patients. In a study involving 225 ICU patients, a significant correlation was found between elevated CPIS scores and the development of HAP, highlighting its clinical utility. This correlation underscores the role of CPIS in facilitating timely medical decision-making by supporting early diagnostic and therapeutic interventions. Early identification of HAP through CPIS enables prompt initiation of appropriate antibiotic treatment, which is crucial in preventing complications-especially those related to multidrug-resistant organisms commonly encountered in intensive care settings. Moreover, the implementation of CPIS as part of routine patient assessment may contribute to improved patient outcomes, reduced duration of ICU stay, and overall enhancement of infection control measures. As a cost-effective and evidence-based scoring system, CPIS plays a vital role in optimizing care for high-risk, critically ill patients.

## Introduction

Hospital-acquired pneumonia (HAP) is defined as pneumonia that occurs 48 hours or more after hospital admission and is a significant contributor to morbidity and mortality, particularly among patients in the intensive care unit (ICU) [1]. A critical subset of HAP is ventilator-associated pneumonia (VAP), prevalent among mechanically ventilated patients, which is associated with extended hospital stays, elevated healthcare costs, and a notably high mortality rate [2]. Additionally, healthcare-associated pneumonia (HCAP) is identified in patients who have frequent interactions with healthcare environments and is often linked to multidrug-resistant pathogens [3].

The incidence of HAP varies between 5 to 20 cases per 1,000 admissions, with key risk factors including advanced age, underlying lung conditions, and the necessity for mechanical ventilation [4]. The Clinical Pulmonary Infection Score (CPIS) (see Appendices) is a valuable tool for the early diagnosis of VAP, evaluating clinical parameters such as body temperature, white blood cell count, tracheal secretions, the PaO_2_/FiO_2_ ratio, and the presence of pulmonary infiltrates [5]. HAP represents 15-20% of hospital-acquired infections, with rates in the ICU escalating to 29%, of which approximately 90% are VAP cases. Mortality associated with VAP is alarmingly high, ranging from 33% to 50%, highlighting its critical nature [6].

The prevalence of HAP is particularly concerning in resource-limited settings, where compromised immune systems heighten vulnerability [7]. Effective management of HAP relies on early detection, stringent infection control measures, and prudent antibiotic use [8]. Integrating preventive strategies, including meticulous hand hygiene, adherence to ventilator care protocols, and antimicrobial stewardship, is pivotal in enhancing patient outcomes and curbing the incidence of hospital-acquired infections [9,10].

HAP is recognized as the second most common hospital-acquired infection, following urinary tract infections, and remains a pressing concern for healthcare professionals despite advancements in therapeutic interventions [11]. The incidence of HAP is particularly pronounced in resource-limited settings, where patients with compromised immune systems are at a distinct disadvantage regarding infection susceptibility [12]. Implementing robust preventive strategies, such as rigorous adherence to hygiene practices, judicious antibiotic stewardship, and careful management of invasive devices, is essential to reducing both HAP and VAP incidence [13].

There is a scarcity of research in tertiary care teaching hospitals, in developing countries like Indian, and studying the impact of socio-demographic factors [14]. The incidences of HAP associated with a distinctive hospital environment are now being identified by emerging research. Comprehensive and multifaceted prevention strategies, the diagnostic capability of CPIS for early detection can add significant value to the healthcare system [15].

The study objective was to assess and correlate the HAP rates among critically ill patients using the CPIS.

## Materials and methods

Research approach and design

This research utilizes a descriptive design to systematically evaluate clinical parameters in patients who are critically ill.

Study setting

The study was carried out in a tertiary care teaching hospital, concentrating on critically ill patients admitted to diverse ICUs.

Population

The target population consists of all patients classified as critically ill, whereas the accessible population is limited to those who were admitted to various ICUs at the designated hospital during the specified study period.

Sample, sample size, and sampling technique

A sample size of 225 patients was calculated using Cochrane’s formula to achieve sufficient statistical power for the study. Participants were selected through a non-probability convenience sampling approach, contingent upon their availability and consent to participate.

Sampling criteria

Inclusion criteria for the study encompassed patients aged 18 years and older who were admitted to the ICU for a minimum duration of 48 hours and showed a willingness to participate during the data collection phase. The exclusion criteria consisted of individuals under 18 years of age, pregnant patients, and individuals who were admitted to non-ICU environments.

Data collection

Data collection was performed using a combination of direct observation and self-reported methods to gather comprehensive clinical information. 

Development of a data collection instrument

Permission was obtained from the creator of the CPIS tool for using it in the study. Pre-testing was conducted on 20 critically ill patients, and reliability was confirmed using the split-half method, yielding a Cronbach’s alpha of 0.829.

Description of the tool

The CPIS was employed to assess the risk of hospital-acquired pneumonia in critically ill patients. Tool A collected demographic information, encompassing variables such as age, gender, admission type, smoking status, oxygen delivery methods, and vital signs. Tool B consisted of six components integral to the CPIS: temperature, white blood cell (WBC) count, characteristics of tracheal secretions, the PaO_2_/FiO_2_ ratio, chest X-ray findings, and results from tracheal cultures. Each component was assigned a score ranging from 0 to 2. A cumulative CPIS score of 7 or higher was indicative of a significant risk for developing ventilator-associated pneumonia (VAP).

Ethical considerations

Ethical approval was obtained from the Institutional Ethics Committee, Mahatma Gandhi Mission's (MGM) Dental College and Hospital, Navi Mumbai (Approval No. MGM/DCH/IEC/132/23, dated April 17, 2023). Institutional permission was also secured from the hospital authority. Informed consent was obtained from each participant prior to data collection to ensure ethical compliance.

Data collection process and plan for data analysis

Data collection commenced following the acquisition of ethical and institutional review board approval. Informed consent was obtained from participants. Analyses were performed using descriptive statistics (frequency and percentage) and inferential statistics (Chi-square tests).

## Results

The clinical data from this patient cohort present a meaningful overview of physiological, radiological, and microbiological parameters relevant to respiratory infections. Most patients (97, 43.1%) had body temperatures within the normal to moderately elevated range, suggesting that fever is not a consistent marker of severe infection in this group. White blood cell (WBC) analysis showed that while 105 (46.7%) exhibited abnormal counts (either leukopenia or leukocytosis), indicating systemic inflammation, 94 (41.8%) maintained normal WBC levels. The presence of bandemia in 26 (11.6%) points to a subset experiencing acute infection, but overall, WBC count alone lacks sufficient predictive value. Tracheal secretions were mild or absent in over half of the patients (121, 53.8%), possibly reflecting early-stage or non-bacterial causes, whereas nearly half showed moderate to purulent secretions, typically associated with infection. A significant majority (199, 88.4%) demonstrated impaired oxygenation, underscoring the widespread presence of pulmonary dysfunction. Radiological assessments revealed that patchy or diffuse infiltrates were more common than localized ones, suggesting a predominance of non-focal lung pathology. Microbiological findings showed limited high bacterial loads (45, 20.0%), and a low rate of Gram stain positivity (15, 6.7%), implying that bacterial pathogens were not the main cause of illness for most. CPIS scores classified 192 (85.3%) as low-risk and 33 (14.7%) as high risk, supporting the overall moderate clinical profile observed (Table 1).

**Table 1:** Analysis of CPIS of the critically ill patients (N=225) ARDS: acute respiratory distress syndrome; CPIS: Clinical Pulmonary Infection Score

Characteristics		f	%
Patient temperature	≥36.5 degrees C and ≤38.4 degrees C	97	43.1
	≥38.5 degrees C and ≤38.9 degrees C	77	34.2
	≥39 degrees C	51	22.7
WBC	<4,000/mm^3^ or >11,000/mm^3^	105	46.7
	<4,000/mm^3^ or >11,000/mm^3^ with ≥500 bands	26	11.6
	≥4,000/mm^3^ and ≤11,000/mm^3^	94	41.8
Tracheal secretions	large and purulent	25	11.1
	Moderate	79	35.1
	No secretion or mild	121	53.8
PaO_2_ (mmHg)/FiO_2_	>240 or ARDS present	26	11.6
	≤240 or no ARDS present	199	88.4
Chest X-ray	localized infiltrate	27	12.0
	No infiltrate	122	54.2
	Patchy or diffuse infiltrate	86	38.2
Quantitative pathogenic bacterial culture growth from tracheal aspirate	>10000	45	20.0
	≤10000 or no growth	165	73.3
	Bacteria seen on Gram stain	15	6.7
CPIS	Low risk	192	85.3
	High risk	33	14.7

The ICU admissions in this study predominantly involve middle-aged adults, with the highest proportion (76, 33.8%) in the 36-45 years age group, followed by those aged 26-35 years (53, 23.6%) and 46-55 years (34, 15.1%). Together, these groups account for over 70% of the patients, emphasizing a significant burden among the economically productive population. Younger adults (16-25 years) and older adults (above 66 years) constitute a smaller share, indicating differing risk or healthcare access patterns across ages. Gender distribution shows a male predominance, with 137 (60.9%) male individuals compared to 88 (39.1%) female individuals, which may reflect occupational hazards or disparities in access to critical care services. Socioeconomically, most patients come from middle-income categories: lower-middle (65, 28.9%), middle (63, 28.0%), and upper-middle (62, 27.6%), suggesting ICU utilization aligns with economic status and healthcare accessibility. Only a small proportion belongs to the high-income (2, 0.9%) and low-income (33, 14.7%) groups. Occupationally, nearly half the patients are employed (102, 45.3%), followed by housewives (67, 29.8%) and retirees (29, 12.9%), demonstrating that ICU admissions span diverse social roles. Lifestyle habits such as smoking or alcohol consumption are reported by 135 (60%) of participants, highlighting modifiable risk factors contributing to critical illness. ICU stay duration predominantly falls between 6-11 days (110, 49%), with shorter stays of 1-5 days (93, 41%), suggesting moderate illness severity or effective clinical management. Clinically, neurological conditions (82, 36.4%) and respiratory diseases (46, 20.4%) are the leading causes of ICU admission, followed by gastrointestinal (30, 13.3%) and cardiovascular (22, 9.8%) disorders (Table 2).

**Table 2:** Distribution of sociodemographic profile of the critically ill patients (N=225)

Characteristics		Frequency	Percentage
Age	16-25	20	8.9
	26-35	53	23.6
	36-45	76	33.8
	46-55	34	15.1
	56-65	18	8.0
	66-75	16	7.1
	76-85	6	2.7
	86-95	2	0.9
Gender	Female	88	39.1
	Male	137	60.9
Socioeconomic status	High: above ₹1,00,000 per month	2	0.9
	Low: below ₹10,000 per month	33	14.7
	Lower-middle: ₹10,000-₹25,000 per month	65	28.9
	Middle: ₹25,000-₹50,000 per month	63	28.0
	Upper-middle: ₹50,000-₹1,00,000 per month	62	27.6
Occupation	Employed	102	45.3
	Housewife	67	29.8
	Retired	29	12.9
	Students	19	8.4
	Unemployed	8	3.6
Any habits (alcohol or smoking)	None	93	41.3
	Yes	135	60.0
ICU stay	1-5 days	93	41
	6-11 days	110	49
	11-15 days	22	10
Diagnosis	Renal system	7	3.1
	Endocrine system	9	4
	Poisoning	11	4.9
	Skeleton system	18	8
	Cardiovascular system	22	9.8
	Gastrointestinal system	30	13.3
	Respiratory system	46	20.4
	Neurological condition	82	36.4

The relationship between the CPIS and the incidence of HAP was assessed using Kendall's tau_b statistical method. This analysis focused on the association between CPIS risk categories (low risk versus high risk) and the corresponding rates of HAP. In the low-risk cohort, out of 151 patients, the vast majority remained free of HAP, with only 41 diagnosed with the infection. Conversely, in the high-risk group, only 12 patients avoided HAP, while 21 acquired the infection. This distribution underscores a distinct trend: higher CPIS correlates with an increased likelihood of developing HAP. The analysis yielded a Kendall’s tau_b coefficient of 0.0335, with a statistically significant p-value (0.001), reinforcing the notion that the CPIS serves as a valid predictive tool for pneumonia risk among hospitalized patients. These findings indicate a direct relationship: as the CPIS risk level rises, so too does the probability of HAP occurrence. This highlights the clinical importance of the CPIS scoring system in stratifying patients who may require intensified monitoring and preventive strategies to mitigate pneumonia risk (Table 3).

**Table 3:** Analysis of correlation of hospital-acquired pneumonia (HAP) rates among critically ill patients with CPIS (N=225) CPIS: Clinical Pulmonary Infection Score

Variable		HAP rates		Correlation (Kendall’s tau_b)	P value
		No	Yes		
CPIS	Low risk	151	41	0.0335	0.001 (significance)
	High risk	12	21		

Statistical analysis via Kendall’s tau_b revealed a significant positive correlation between CPIS and the incidence of HAP (τ_b = 0.0335, p = 0.001), underscoring the role of CPIS as an early predictor of infection. While the score demonstrated high sensitivity (89%), its specificity was moderate (47%), suggesting that more than half of the patients with elevated CPIS values could be subjected to unwarranted antibiotic therapy in the absence of confirmed infection.

## Discussion

The CPIS synthesizes various clinical and laboratory parameters, including body temperature, white blood cell count, oxygenation status (PaO_2_/FiO_2_ ratio), tracheal secretions, chest radiograph findings, and microbiological culture results, to yield a composite score spanning 0 to 12 [1]. A CPIS threshold above 6 is broadly endorsed as indicative of a high likelihood of HAP [16].

In a cohort of 201 critically ill patients (mean age 44.2 years; 61% male), CPIS evaluations conducted on day 3 indicated that 69% exhibited scores surpassing this diagnostic threshold. Notably, bronchoscopy paired with microbiological verification confirmed HAP in only 44% of these instances, resulting in a kappa coefficient of 0.33, which denotes moderate agreement between CPIS findings and bronchoscopic diagnoses [3]. This discordance is crucial for clinical decision-making.

These findings align with foundational work by Pugin et al. (1991), who initially proposed the CPIS and established that scores exceeding 6 were predictive of ventilator-associated pneumonia [16]. This study not only reaffirms these earlier observations but also presents updated demographic and clinical data, including socioeconomic factors and the prevalence of risk factors (notably, 58.7% with a history of smoking or alcohol use) [8,9].

Given the CPIS’s limited specificity, our results advocate for the integration of confirmatory diagnostic modalities, such as bronchoscopy, quantitative cultures, or advanced imaging, aimed at refining diagnostic accuracy and mitigating unnecessary antimicrobial exposure. Implementing the CPIS within ICU protocols can enable earlier therapeutic interventions, potentially lowering the incidence of severe complications like sepsis and respiratory failure. However, a judicious, evidence-based approach remains critical to optimizing patient management and advancing antimicrobial stewardship in critically ill populations.

Limitations

The data collection period was confined to two months, resulting in a relatively modest sample size. Additionally, the absence of a control group restricted the study to a purely descriptive methodology. Furthermore, the sample was drawn exclusively from a single hospital, which may limit the generalizability of the findings. The study was conducted only on adult patients who were admitted to the ICU.

## Conclusions

The CPIS serves as a valuable tool for the early identification and management of HAP in critically ill patients, particularly within tertiary care teaching hospitals. Designed to streamline the diagnostic process, CPIS evaluates a combination of clinical, radiological, and laboratory parameters, such as temperature, white blood cell count, oxygenation, and chest radiograph findings. This structured assessment enables healthcare professionals to make timely and informed decisions regarding the presence and severity of HAP. CPIS provides a proactive approach to support clinical judgment, reduce diagnostic uncertainty, and guide appropriate therapeutic interventions. By incorporating CPIS into routine clinical workflows, care teams can improve response times, standardize assessment practices, and potentially reduce the incidence of complications associated with delayed treatment. Along with clinical expertise, CPIS complements definitive diagnostic methods for infection control.

Overall, CPIS represents a strategic advancement in the ongoing effort to improve healthcare quality in critical care environments. Its use fosters earlier recognition of HAP, supports prompt medical intervention, and aligns with broader goals of improving patient outcomes and minimizing hospital-acquired infections. As part of a comprehensive clinical strategy, CPIS can be a key component in enhancing care delivery and addressing the challenges associated with managing complex infections in vulnerable patient populations.

## Appendices

**Table 4:** Clinical Pulmonary Infection Score (CPIS) ARDS: acute respiratory distress syndrome Interpretation of the tool: minimum score = 0; maximum score = 12; score>6 tells there is a maximum chances of developing infection Credits: Jerome Pugin Permission for the CPIS tool [16] was taken from the tool creator for using the tool

Criteria	Points
Patient temperature	
≥36.5 degrees C and ≤38.4 degrees C	0
≥38.5 degrees C and ≤38.9 degrees C	1
≥39 degrees C	2
WBC	
≥4,000/mm^-3^ and ≤11,000/mm^-3^	0
<4,000/mm^-3^ or >11,000/mm^-3^	1
<4,000/mm^-3^ or >11,000/mm^-3^ with ≥500 bands	2
Tracheal secretions	
No secretion or mild	0
Moderate	1
large and purulent	2
PaO_2 _(mmHg)/FiO_2_	
>240 or ARDS present	0
≤240 or no ARDS present	2
Chest X-ray	
No infiltrate	0
Patchy or diffuse infiltrate	1
Localized infiltrate	2
Quantitative pathogenic bacterial culture growth from tracheal aspirate	
≤10000 or no growth	0
>10000	1
Bacteria seen on Gram stain	2
